# Sorption-enhanced gasification of municipal solid waste for hydrogen production: a comparative techno-economic analysis using limestone, dolomite and doped limestone

**DOI:** 10.1007/s13399-022-02926-y

**Published:** 2022-06-23

**Authors:** Mónica P. S. Santos, Dawid P. Hanak

**Affiliations:** Energy and Power, School of Water, Energy and Environment, Cranfield University, Bedford, Bedfordshire MK43 0AL UK

**Keywords:** Sorption-enhanced gasification, Waste-to-fuel, Hydrogen production, Dolomite, Doped limestone

## Abstract

Sorption-enhanced gasification has been shown as a viable low-carbon alternative to conventional gasification, as it enables simultaneous gasification with in-situ CO_2_ capture to enhance the production of H_2_. CaO-based sorbents have been a preferred choice due to their low cost and wide availability. This work assessed the technical and economic viability of sorption-enhanced gasification using natural limestone, doped limestone with seawater and dolomite. The techno-economic performance of the sorption-enhanced gasification using different sorbents was compared with that of conventional gasification. Regarding the thermodynamic performance, dolomite presented the worst performance (46.0% of H_2_ production efficiency), whereas doped limestone presented the highest H_2_ production efficiency (50.0%). The use of dolomite also resulted in the highest levelised cost of hydrogen (5.4 €/kg against 5.0 €/kg when limestone is used as sorbent), which translates into a CO_2_ avoided cost ranging between 114.9 €/tCO_2_ (natural limestone) and 130.4 €/tCO_2_ (dolomite). Although doped limestone has shown a CO_2_ avoided cost of 117.7 €/tCO_2_, this can be reduced if the production cost of doped limestone is lower than 42.6 €/t. The production costs of new sorbents for CO_2_ capture and H_2_ production need to be similar to that of natural limestone to become an attractive alternative to natural limestone.

**Supplementary Information:**

The online version contains supplementary material available at 10.1007/s13399-022-02926-y.

## Introduction

Global carbon dioxide (CO_2_) emissions have been rising for over a century now [1]. Although a significant emission reduction was recorded in 2021, mostly due to reduced economic activity caused by COVID-19, the global energy-related CO_2_ emissions bounced back to the pre-pandemic levels in 2021 [2]. Thus, unless the CO_2_ and other greenhouse emissions are significantly reduced, the 1.5 °C and 2 °C global warming scenarios will be overcome until the end of this century [3]. Carbon capture and storage (CCS), as well as the reduction of fossil fuel dependency, have been identified as routes to tackle CO_2_ emissions. The latter can be achieved by expanding the production of cleaner fuels and energy carriers, including hydrogen.

Hydrogen (H_2_) or H_2_-rich syngas production has been thoroughly investigated from different types of biomass and wastes feedstocks such as sawdust [4], sewage sludge [5, 6], hazelnut shells [7], wood chips [8], wood pellets [9], palm kernel shell [10], plastics [11], food waste [12] and municipal solid waste (MSW) [13]. Although biomass is a renewable source and accessible at a reduced price, the generalised use of biomass for bioenergy production can start competing with food and crops production. Thus, wastes from agriculture, landfills, food, biomass residues, sewage sludge and manure, are regarded as sustainable feedstocks and should play a role in decarbonisation [14].

Considering the current state of solid waste management, there are significant differences between developed and developing countries, with the latter relying on open waste dumping [15]. Such practice, on top of open burning and unsanitary landfills, raises several environmental issues comprising global warming, ozone and resources depletion, damage of ecosystems and human health hazards [16]. Furthermore, it is forecasted that the annual CO_2,eq_ emissions associated with solid wastes can reach 2.6 billion tonnes by 2050 if no improvements in waste management are deployed. It is because the amount of solid waste generated by developing countries is forecasted to triple by that year [15].

A range of technologies for thermochemical conversion of MSW have been considered, including plasma gasification [17, 18], chemical looping combustion [19], gasification [20, 21], gasification integrated with simultaneous chemical and calcium looping [22], and sorption-enhanced gasification (SEG) [13, 23–27]. MSW SEG was assessed by He et al. [23] in a lab-scale fixed bed reactor at the gasification temperature of 900 °C. They have investigated the catalytic effect of calcined dolomite on the gasification performance, as well as the effect of the steam/MSW ratio on the gas composition and H_2_ yield. They found that the increase in a steam/MSW ratio increased the H_2_ mole fraction and the H_2_ yield, which peaked (53% and 43 mol H_2_/kg MSW, respectively) for a steam/MSW ratio of 1.04. Hu et al. [24] used CaO as a CO_2_ sorbent to study the effect of parameters such as gasification temperature, Ca/C molar ratio and moisture content on the H_2_ yield and syngas composition. The experiments were carried out in a lab-scale fixed bed reactor. They found that a maximum H_2_ mole fraction in syngas (49.4%) could be achieved at the gasification temperature of 750 °C, a CaO/MSW molar ratio of 0.7 and moisture content in MSW of 40%. Similarly, Zhou et al. [25] selected a fixed bed to study the effect of CaO sorbent on the performance of MSW steam SEG. In that case, a lab-scale batch type reactor was used. The H_2_ mole fraction in the syngas has been shown to increase by 15% points with the addition of CaO sorbent, from around 35% when no sorbent was present in the reactor. This result was achieved for a CaO/MSW mass ratio of 1 and at the gasification temperature of 700 °C. They have also shown that CaO acts as a CO_2_ sorbent and catalyst, being responsible for enhancing the MSW devolatilization and char gasification. The use of waste marble powder as a CaO-based sorbent and simultaneously a catalyst was investigated by Irfan et al. [13] in a lab-scale batch-type reactor. The performance of MSW SEG, including syngas yield and composition, tar content and carbon conversion efficiency, were evaluated at different gasification temperatures, steam/MSW and sorbent/MSW ratios. They found that the increase in gasification temperature, steam/MSW and sorbent/MSW ratios promoted the H_2_ mole fraction in the syngas. Consequently, the syngas yield and the carbon conversion efficiency have increased. However, it is important to notice that an increase in the considered operating conditions also resulted in a decrease in the tar formation. The concept of MSW SEG has also been proven to be a feasible technology for H_2_-rich gas production at a 30 kW_th_ bubbling fluidised bed (BFB) plant [26]. This study considered limestone as a CO_2_ sorbent, mostly because of its availability and low cost. Yet, this study did not consider the deterioration of the sorbent performance, which is a known challenge of using limestone as a sorbent [28], because the set-up was operated in a semi-batch mode. Finally, Santos et al. [27] have compared the techno-economic performance of MSW steam SEG with that of conventional steam gasification for H_2_ production. The authors have shown that MSW SEG could deliver a higher H_2_ production efficiency (48.7%) than conventional gasification (47.7%). Yet, such improvement is obtained at the expense of higher H_2_ production costs, the levelised cost of H_2_ (*LCOH*) increased from 2.1–3.2 €/kg H_2_ (conventional gasification) to 4.5–5.1 €/kg H_2_ (SEG).

Natural materials such as the shells from mollusc, scallop, oyster and mussel as well as eggshells have been shown viable as a CO_2_ sorbent. However, the calcined sorbent also presented deactivation along with the carbonation/calcination cycles [29]. Moreover, it would be challenging to convert such waste to sorbent for large-scale applications. Thus, alternative sorbents for SEG need to be considered. These sorbents, besides the high CO_2_ sorption capacity, should also present fast sorption kinetics, good mechanical properties, good cyclic stability and be economically viable [30]. If the properties of synthetic sorbents are easier to be manipulated, the high cost of the chemical precursors results in a higher cost of sorbent production [31].

Therefore, several approaches have been considered to enhance the performance of natural CaO-based sorbents, including the incorporation of inert materials with high Tammann temperatures, doping of sorbent and additional treatments including hydration or chemical pretreatment.

Several inert materials have been studied as potential support materials to improve the sorbent stability, including aluminium oxide (Al_2_O_3_) [32, 33], magnesium oxide (MgO) [33], zirconium oxide (ZrO_2_) [33, 34], titanium oxide (TiO_2_) [35], yttrium oxide (Y_2_O_3_) [33], and silica (SiO_2_) [36]. Because these materials modify the sorbent skeleton, the sorbent granulation is improved and the sintering phenomenon is prevented. However, these sorbents are more expensive than natural CaO-based sorbents.

Hydration is another technique investigated in the current literature to increase the sorption capacity of the CaO-based sorbents. Water hydration [37] or steam hydration [38] can be used to improve the CO_2_ sorption capacity of the fresh sorbent. This technique has also been applied for reactivation of the spent sorbent [39]. In the latter case, the enhancement of sorption capacity is attributed to the formation of calcium hydroxide (Ca(OH)_2_). Since the molecule Ca(OH)_2_ presents a higher molar volume than CaO, this contributes to the formation of cracks and then paths. This morphology alteration increases the surface area and pore volume, enhancing the CO_2_ sorption [40].

The chemical treatment is another approach considered in the current literature to improve the CO_2_ sorption capacity of the sorbent. In this approach, an enhancement of sorption capacity is achieved by treating the sorbent with chemicals such as acetic acid and pyroligneous acid [30]. Li et al. [41] have found that after 20 cycles of carbonation/calcination, the conversion of limestone pretreated with acetic acid increased by more than a factor of 3 when compared with that of natural limestone (0.5 against 0.15). This can be attributed to the higher surface area and higher pore volume of treated limestone, which prevents the sintering phenomenon.

The CO_2_ sorption capacity of sorbent has been shown to be enhanced by doping the CaO-based sorbents with sodium chloride (NaCl) [42], hydrogen bromide (HBr) [43] and seawater [44, 45]. Salvador et al. [42] have studied the effect of doping limestone with 0.5%_wt_ NaCl in a thermogravimetric analyser (TGA) and fluidised bed reactor. While in the tests performed in the fluidised bed reactor, there was no positive effect on the CO_2_ sorption capacity. In the TGA experiments, this figure was higher than that for natural limestone after 14 cycles. It should be noted that in the first cycles the natural limestone presented a better performance than the doped one. The addition of 0.167%_mol_ HBr to limestone was studied by González et al. [43] in a fluidised bed reactor. The authors found that the CO_2_ sorption capacity of limestone, after 13 cycles, doubled when compared with the natural limestone. Xu et al. [44] have investigated the possibility of using an abundant and cheap material, seawater, as a dopant to improve the CO_2_ sorption capacity of limestone. They have carried out 20 cycles of carbonation/calcination in a fixed bed reactor. The authors concluded the CO_2_ sorption capacity was maximum for 0.25%_wt_ of dopant. Morona et al. [46] have also studied the doping of limestone with different concentrations of seawater. After the sorbent had undergone 20 cycles of carbonation/calcination, the carbonation conversion was evaluated in a TGA. Unlike the previous work, the authors observed a deleterious effect on sorbent performance when dopped with seawater, which can be associated with an excessive addition of dopant [47]. The use of seawater as a dopant was also studied by González et al. [45], although in this work, the experiments were performed in a fluidised bed reactor. Similarly, to previous work, the authors have investigated different concentrations of dopant. They concluded that the addition of seawater to the four limestones tested improved the sorbent performance.

Similar to limestone, dolomite is another inexpensive natural CaO-based sorbent available worldwide. De La Calle Martos et al. [48] have compared the limestone and dolomite CO_2_ capture performance in a TGA, undergoing 20 cycles of carbonation/calcination. They concluded that dolomite compared with limestone presented the following advantages: lower regeneration temperature, lower deactivation along with the cycles, superior CaO conversion, and thus, a higher CO_2_ capture capacity. Although dolomite and doped limestone have been extensively assessed, the mentioned studies were carried out from a CO_2_ capture performance standpoint of view. Moreover, the majority of studies that assessed SEG to date have solely focused on syngas production through MSW gasification with in-situ CO_2_ capture, disregarding the sorbent regeneration step that is essential for the continuous operation of the SEG process. Santos and Hanak [27] have reported for the first time the techno-economic feasibility of a cyclic SEG of MSW for H_2_ production. However, that study only assessed the H_2_ production costs for SEG of MSW using natural limestone as sorbent and, therefore, no comparative study has been carried out for other sorbents. Martínez et al. [26] and Santos and Hanak [27] have shown that SEG using limestone is a feasible technology to convert waste-to-fuel, despite the fact that the economic assessment performed by Santos and Hanak [27] has shown it is still not competitive. Yet, dolomite and doped limestone may be an attractive alternative to natural limestone sorbent, promoting the deployment of SEG of MSW. Dolomite presents higher CO_2_ desorption kinetics, which implies lower calcination temperature and, thus, a lower energy penalty. Doped limestone has shown to have a lower reactivity decaying over the cycles, improving CO_2_ sorption capacity and, therefore, leading to an enhancement of H_2_ production. Thus, it is crucial to evaluate these alternative sorbents for H_2_ production with in-situ CO_2_ capture.

This work aims to examine whether alternative sorbents can improve the techno-economic viability of MSW SEG for H_2_ production. The techno-economic assessment of the MSW SEG was performed for three different CaO-based sorbents, including natural limestone, doped limestone with seawater and dolomite. Furthermore, an assessment of the MSW SEG design specifications and economic assumptions on the process energy, economic and environmental performance was assessed via a sensitivity analysis.

## Process and model description

**Fig. 1:**
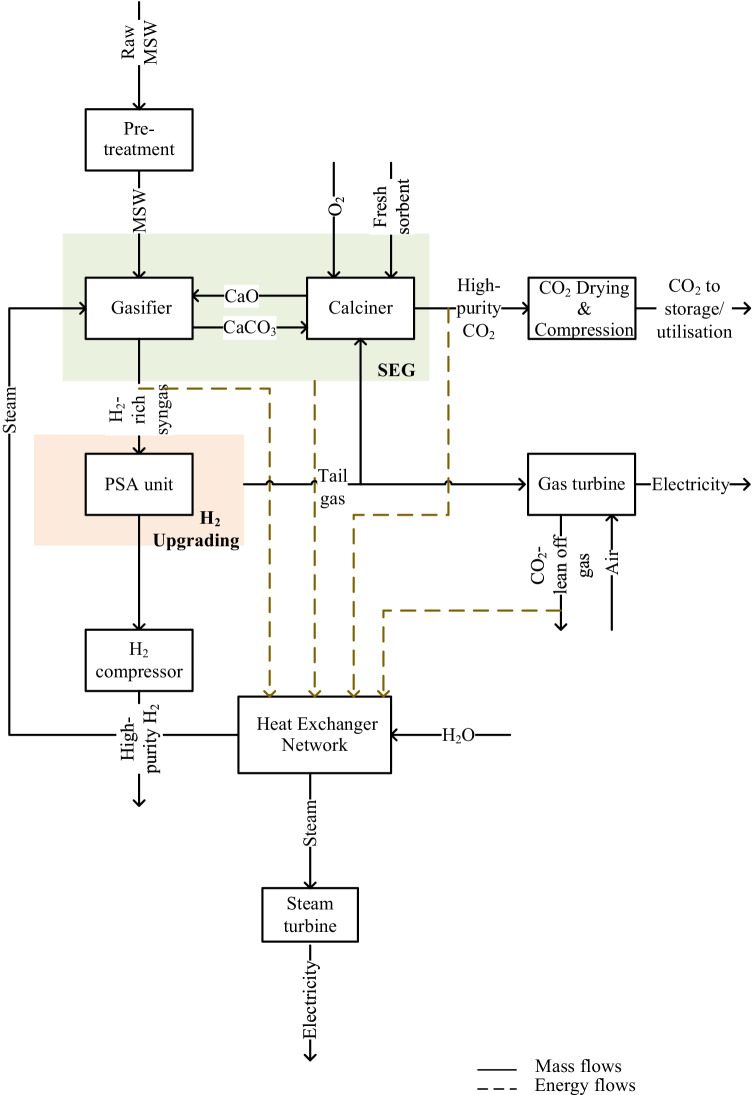
Simplified block diagram representation of sorption-enhanced gasification of municipal solid waste for hydrogen production

MSW, whose characteristics are shown in Table 1, was selected as feedstock for hydrogen production through SEG. The MSW SEG process is presented in the simplified block flow diagram in Fig. 1. The operating conditions are listed in Table 2. The MSW processing rate was assumed to be 500 t of MSW per day, corresponding to approximately 100 MW_th_. The SEG process model, based on a set of mass and energy balances, was developed in Aspen Plus. The model was based on the Gibbs free energy minimisation approach. The physical properties of the components were assessed using the Peng-Robinson equation of state with Boston-Mathias modifications. To simplify the model, it was considered that: (1) the process is isothermal, (2) the process operates under steady-state conditions, (3) the heat losses and the pressure drops are negligible, (4) graphitic carbon is the only compound of char, (5) ash is inert and (6) the formation of tar and higher hydrocarbon is negligible. The SEG process was validated with the experimental data reported by Fremaux et al. [49] and Armbrust et al. [50]. The syngas composition was obtained at 700 °C, 800 °C and 900 °C for a range of *SBR* between 0.5 and 1.0, besides the H_2_ yield obtained at the same temperatures and for the *SBR* 0.5, 0.7 and 1.0 were compared with that obtained by the model. The experiments carried out by Armbrust et al. [50] at two different syngas compositions were used to validate the carbonation reaction and thus the H_2_-rich gas composition. Besides the syngas composition, the carbonation temperature (637 °C and 643 °C) and sorbent looping ratio (7.0 and 8.6) were used to validate the process. The SEG process validation is described in detail by Santos and Hanak [27].

**Table 1:** Municipal solid waste properties [52]

Proximate analysis [%_wt_ db]	
Ash	7.12
Fixed carbon	15.36
Volatile matter	77.52
Ultimate analysis [%wt db]	
Carbon	49.51
Oxygen	35.69
Hydrogen	6.42
Nitrogen	0.78
Sulphur	0.48
Moisture [%_wt_]	9.34
LHV [MJ/kg]	19.99

*db* dry basis, *LHV* lower heating value

**Table 2:** Summary of the key sorption-enhanced gasification model assumptions

Unit operation	Parameter	Value
Sorption-enhanced gasification		
Sorption-enhanced gasifier	Temperature (°C)	650
	Steam-to-biomass ratio (wt/wt)	0.5–1.7 (dolomite)
		0.5–2.0 (limestone and doped limestone)
	Carbonation extent (-)	0.7
	CO_2_ capture efficiency in carbonator (%)	90.0
Calciner	Temperature (°C)	850 (dolomite)
		900 (limestone and doped limestone)
	Calcination extent (-)	0.95
	Excess oxygen (%_vol,dry_)	2.5
	Ratio between fresh make-up sorbent rate and sorbent circulation rate (-)	0.02
H_2_-rich syngas upgrading		
Compression		
Compressor	Polytropic efficiency (%)	80.0
	Mechanical efficiency (%)	99.6
H_2_-rich syngas final stream	Temperature (°C)	30
	Pressure (bar)	34
PSA	H_2_ recovery (%)	93.0
	H_2_ purity (%_vol_)	99.9
	Temperature (°C)	30
	Feed pressure (bar)	34
	Tail gas pressure (bar)	1
	Delivery pressure (bar)	60
CO_2_ compression		
Compressors	Polytropic efficiency (%)	80.0
	Mechanical efficiency (%)	99.6
Pump	Isentropic efficiency (%)	80.0
	Mechanical efficiency (%)	99.6
CO_2_ final stream	Temperature (°C)	25.0
	Pressure (bar)	110.0
Steam cycle		
Condenser	Fresh water temperature (°C)	10.0
Low-pressure turbine	Isentropic efficiency (%)	88
	Mechanical efficiency (%)	98
Intermediate-pressure turbine	Isentropic efficiency (%)	94
	Mechanical efficiency (%)	99.8
High-pressure turbine	Isentropic efficiency (%)	92.0
	Mechanical efficiency (%)	99.8
Live steam	Temperature (°C)	593.0
	Pressure (bar)	154.0
Gas turbine		
	Turbine inlet temperature (°C)	1268
	Turbine isentropic efficiency (%)	80
	Turbine mechanical efficiency (%)	99.6
	Compressor outlet pressure (bar)	20
	Combustor pressure drop (%)	2
Fresh material [59]	Dolomite (57.5%_wt_ CaCO_3_, 42.44%_wt_ MgCO_3_, 0.01%_wt_ SiO_2_, 0.02%_wt_ Fe_2_O_3_, 0.03%_wt_ Al_2_O_3_)	
	Limestone (95.0%_wt_ CaCO_3_, 3.5%_wt_ MgCO_3_, 0.6%_wt_ SiO_2_, 0.4%_wt_ Fe_2_O_3_, 0.5%_wt_ Al_2_O_3_)	

Since the raw MSW is not suitable for gasification, this is subjected to a pretreatment [51]. In the first stage, the recyclables and non-recyclables are separated (primary separation). In the second stage, mechanical treatment is carried out to produce briquettes suitable to be gasified. Therefore, the energy requirement and the costs of pretreatment have been accounted for in the techno-economic evaluation. The data detailed by Luz et al. [51] was used to appraise both.

As shown in Fig. 1, the MSW SEG plant comprises a gasifier operating in parallel with a calciner. In this work, three CaO precursor sorbents were selected, natural limestone, dolomite and doped limestone with seawater. In the SEG process, sorbent acts as a heat and CO_2_ carrier, and circulates between the two interconnected fluidised beds. The CO_2_ removal by the sorbent takes place in a gasifier (Eq. (1)), whereas the sorbent regeneration, represented by Eqs. (2) and (3), takes place in a calciner. It should be noted that Eq. (3) corresponds to the first stage of dolomite decomposition, which occurs at around 700 °C and is not dependent on the CO_2_ content in the gas phase present in the calciner [48].

Carbonation:

1 $$\text{CaO}\left(\mathrm{s}\right)+\mathrm{C}{\mathrm{O}}_2\left(\mathrm{g}\right)\to \text{CaC}{\mathrm{O}}_3\left(\mathrm{s}\right)\kern12.5em \Delta H=-178\ \text{kJ}/\text{mol}$$

Calcination:

2 $$\text{CaC}{\mathrm{O}}_3\left(\mathrm{s}\right)\to \text{CaO}\left(\mathrm{s}\right)+\mathrm{C}{\mathrm{O}}_2\left(\mathrm{g}\right)\kern13.25em \Delta H=178\ \text{kJ}/\text{mol}$$

Dolomite decomposition:

3 $$\text{MgCa}{\left(\mathrm{C}{\mathrm{O}}_3\right)}_2\left(\mathrm{s}\right)\to \text{CaC}{\mathrm{O}}_3\left(\mathrm{s}\right)+\text{MgO}\left(\mathrm{s}\right)+\mathrm{C}{\mathrm{O}}_2\left(\mathrm{g}\right)\kern7.25em \Delta H=127\ \text{kJ}/\text{mol}$$

To compensate for the sorbent deactivation and corresponding decrease in the CaO conversion, a fresh stream of sorbent, called make-up (*F*_0_), is fed to the calciner. Equation (4) represents the maximum average conversion (*X*_ave_) that can be accomplished by the sorbent over the cycles of carbonation/calcination and is based on the model presented by Rodríguez et al. [28]. The maximum average conversion depends on the properties of sorbent (*a*_*1*_, *a*_2_, *f*_1_, *f*_2_ and *b*), the carbonated (*f*_carb_) and calcined sorbent fraction (*f*_calc_), the fresh make-up sorbent rate (*F*_0_) and the sorbent circulation rate (*F*_R_). The sorbent properties were determined by the curve-fitting procedure. The experimental data detailed by Zhen-Shan et al. [53] and González et al. [45] were used to determine the sorbent characteristics for dolomite and doped limestone, respectively. It is noteworthy that since the gasification and CO_2_ capture take place in the gasifier, the solid stream leaving the gasifier comprises the sorbent and the ash. Part of the ash is purged with the deactivated sorbent after the calcination.

4 $${X}_{\text{ave}}=\left({F}_0+{F}_R{r}_0\right){f}_{\text{calc}}\left[\frac{a_1{f}_1^2}{F_0+{F}_R{f}_{\text{carb}}{f}_{\text{calc}}\left(1-{f}_1\right)}+\frac{a_2{f}_2^2}{F_0+{F}_R{f}_{\text{carb}}{f}_{\text{calc}}\left(1-{f}_2\right)}+\frac{b}{F_0}\right]$$

The heat required by the endothermic calcination reaction is met by the oxy-combustion of the unconverted char and a fraction of the tail gas from the H_2_ upgrading unit. It was assumed that the O_2_ is delivered by a cryogenic air separation unit (ASU), which corresponds to energy consumption of 200 kW_el_h/tO_2_ [54]. Since the gasification and CO_2_ capture take place simultaneously in the same reactor, the equilibrium of water gas shift reaction, Eq. (5), is altered and the forward reaction is favoured, enhancing the H_2_ formation. Moreover, this kind of integration is beneficial because the heat released by the exothermic carbonation reaction and the sensible heat of sorbent sustains the endothermic gasification process.

Water gas shift:

5 $$\mathrm{C}\mathrm{O}\left(\mathrm{g}\right)+{\mathrm{H}}_2\mathrm{O}\left(\mathrm{g}\right)\leftrightarrow \mathrm{C}{\mathrm{O}}_2\left(\mathrm{g}\right)+{\mathrm{H}}_2\left(\mathrm{g}\right)\kern9em \Delta H=\pm 40.9\ \text{kJ}/\text{mol}$$

Two reactors, Gibbs reactor and stoichiometric reactor, were used to represent the gasification and carbonation processes, respectively. As these processes occur in the same reactor, a heat stream is connected between the Gibbs and stoichiometric reactors. A Gibbs reactor was also used to model the calcination process. It should be noted that besides the reactor units of SEG, a pressure swing adsorption (PSA) unit, an ASU, a gas turbine, a heat exchanger network and a CO_2_ compression unit are also components of the plant. Because the temperature of flue gas leaving the combustor chamber is close to the adiabatic flame temperature, this is mixed with compressed air at 20 bar to lower the turbine inlet temperature to 1268 °C. Then, the flue gas is expanded in the turbine that is coupled with the generator to produce electricity. It was assumed that the H_2_-rich stream produced in the sorption-enhanced gasifier is upgraded in a PSA unit, which was modelled as a black box. According to Luberti et al. [55] and Hu [56], an H_2_ stream with 99.9% of purity and 93% of recovery rate is achievable at 34 bar. Consequently, the H_2_-rich syngas produced in the sorption-enhanced gasifier is compressed from 1 bar to 34 bar in a 9-stage compressor. It should be noted the H_2_-rich stream upgrading is preceded by cooling the gas to 30 °C and water removal stages. The heat recovered during these stages is integrated into the heat exchanger network to produce steam that is used as the gasifying agent in the SEG process. Then, the high-purity H_2_ stream is subjected to compression until 60 bar. Part of the tail gas from the PSA unit is used to meet the calciner energy requirement and the remaining part is burnt in the gas turbine to generate electricity. In the CO_2_ compression unit, the pressure of the high-purity CO_2_ stream produced in the calciner is increased to 110 bar and the temperature is reduced to 25 °C [57]. The high-grade heat of the high-purity CO_2_ stream, along with the one from the CO_2_-lean off-gas, is recovered in the heat exchanger network and used in the steam cycle to produce electricity. The SEG model, as well as the steam cycle, have been described in detail and validated elsewhere [27, 58].

## Techno-economic feasibility assessment

In this work, the techno-economic analysis of MSW SEG for dolomite and doped limestone was evaluated at its best performance to ensure a fair comparison. As explained in Sect. 4.1, it was assumed that the best performance of each sorbent is achieved for the highest value of *SBR* at which the plant is energy self-sufficient. These figures were benchmarked with that obtained for SEG using limestone as sorbent and conventional gasification [27].

### Thermodynamic performance indicators

H_2_ production efficiency, gross power efficiency, net power efficiency and total efficiency were the indicators chosen to appraise and compare the thermodynamic performance of SEG and conventional gasification. The H_2_ production efficiency, described by Eq. (6), is the coefficient between the heat content of the product, H_2_ and the fuel heat content, MSW. ${LHV}_{H_2}$ and ${\dot{m}}_{H_2}$ represent the lower heating value and mass flow rate of H_2_, respectively, and *LHV*_*MSW*_ and ${\dot{m}}_{MSW}$ correspond to the same variables for MSW.

6 $${\upeta}_{{\mathrm{H}}_2}=\frac{{\dot{\mathrm{m}}}_{{\mathrm{H}}_2}\bullet {\text{LHV}}_{{\mathrm{H}}_2}}{{\dot{\mathrm{m}}}_{\text{MSW}}\bullet {\text{LHV}}_{\text{MSW}}}$$

The ratio between the sum of electric power output from the gas turbine and the steam cycle (*W*_el,gross_) and the fuel heat content, MSW, defines the gross power efficiency (*η*_el,gross_) defined by Eq. (7).

7 $${\upeta}_{\text{el, gross}}=\frac{{\mathrm{W}}_{\text{el, gross}}}{{\dot{\mathrm{m}}}_{\text{MSW}}\bullet {\text{LHV}}_{\text{MSW}}}$$

Equation (8) represents the net power efficiency (*W*_el,net_) that is defined as the ratio between the net electric power output (*W*_el,net_) and the fuel heat content, MSW. The former is the difference between the gross electric power output and the electric power demand by the auxiliary equipment.

8 $${\upeta}_{\text{el, net}}=\frac{{\mathrm{W}}_{\text{el, net}}}{{\dot{\mathrm{m}}}_{\text{MSW}}\bullet {\text{LHV}}_{\text{MSW}}}$$

The sum of H_2_ production and net power efficiencies, defines the total efficiency (*η*_tot_) represented by Eq. (9).

9 $${\upeta}_{\text{tot}}=\frac{\left({\dot{\mathrm{m}}}_{{\mathrm{H}}_2}\bullet {\text{LHV}}_{{\mathrm{H}}_2}\right)+{\mathrm{W}}_{\text{el, net}}}{{\dot{\mathrm{m}}}_{\text{MSW}}\bullet {\text{LHV}}_{\text{MSW}}}$$

### Economic performance indicators

The *LCOH* and the cost of CO_2_ avoided (*AC*) were selected as indicators to assess the economic performance of SEG. These were used to benchmark the performance of MSW SEG with that of conventional gasification. The *LCOH*, minimum H_2_ selling price at which the profits offset the total costs over the project lifetime, was estimated based on the net present value (*NPV*). The capital costs of each piece of equipment were scaled up using a scaling size factor. All the correlations are listed as supplementary information. The approach used to assess the total capital requirement is detailed in Santos and Hanak [27]. Although the inflation was not considered over the project lifetime, the capital costs were updated to the year 2017 using Chemical Engineering Plant Cost Index (CEPCI) [60].

Since in this work all the costs are presented in Euro (€), if the costs reported in the literature were in a different currency, an average conversion rate for the year 2017 was used [61]. The average conversion rate for the year 2017 and the other economic parameters and assumptions are summarised in Table 3. The operating and maintenance costs account for the variable and the fixed costs. The former was calculated based on the production output, including the costs associated with raw materials, utilities and CO_2_ transport and storage. To estimate the latter, it was assumed 17.8% of the total capital requirement is spent to cover the costs associated with salaries, insurance and tax payments, mortgage payments and indirect expenses of running a business [8].

**Table 3:** Parameters used to assess the economic performance

Parameter	Value
Unit cost of electricity exported to the grid (€/MW_el_h) [62]	40.0
Limestone unit cost (€/t) [8]	11.6
Dolomite unit cost (€/t)	11.6
Doped limestone unit cost (€/t)	58.0^a^
Fresh water unit cost (€/m^3^) [8]	2.4
CO_2_ transport and storage cost (€/t) [63]	20.0
Others	
Project interest rate (%) [64, 65]	8.8
Project lifetime (y) [64, 65]	25.0
Capacity factor (%) [64, 65]	80.0
Average GBP/EUR exchange rate 2017 [61]	1.1418
Average USD/EUR exchange rate 2017 [61]	0.8898
CO_2_ emission allowance price (€/tCO_2_) [66]	39.6
Gate fee (€/t_MSW_) [67]	40.0

^a^The price of doped limestone was assumed to be 5 times the price of natural limestone (11.6 €/t) to account the doping and drying of sorbent

The cost of CO_2_ avoided, given by Eq. (10), is the ratio between the difference of *LCOH* and the difference of equivalent CO_2_ emissions ${e}_{\mathrm{C}{\mathrm{O}}_2,\text{eq}}$ of conventional gasification and of sorption-enhanced gasification plants. The equivalent CO_2_ emission accounts for the direct and indirect CO_2_ emissions. The latter is associated with the electric power imported or exported by the plant. The subscripts Gasf and SEG refer to conventional gasification and sorption-enhanced gasification, respectively.

10 $$AC=\frac{LCOH_{\text{SEG}}-\kern0.5em {LCOH}_{\text{Gasf}}}{e_{\mathrm{C}{\mathrm{O}}_2,\text{eq, Gasf}}-\kern0.5em {e}_{\mathrm{C}{\mathrm{O}}_2,\text{eq, SEG}}}$$

## Results and discussion

The techno-economic performance of SEG using dolomite and doped limestone with seawater as sorbent was assessed based on the indicators defined in the previous section. These sorbents’ performance was compared with that obtained for SEG using natural limestone. The SEG performance was benchmarked with the conventional gasification.

### Thermodynamic performance

**Fig. 2:**
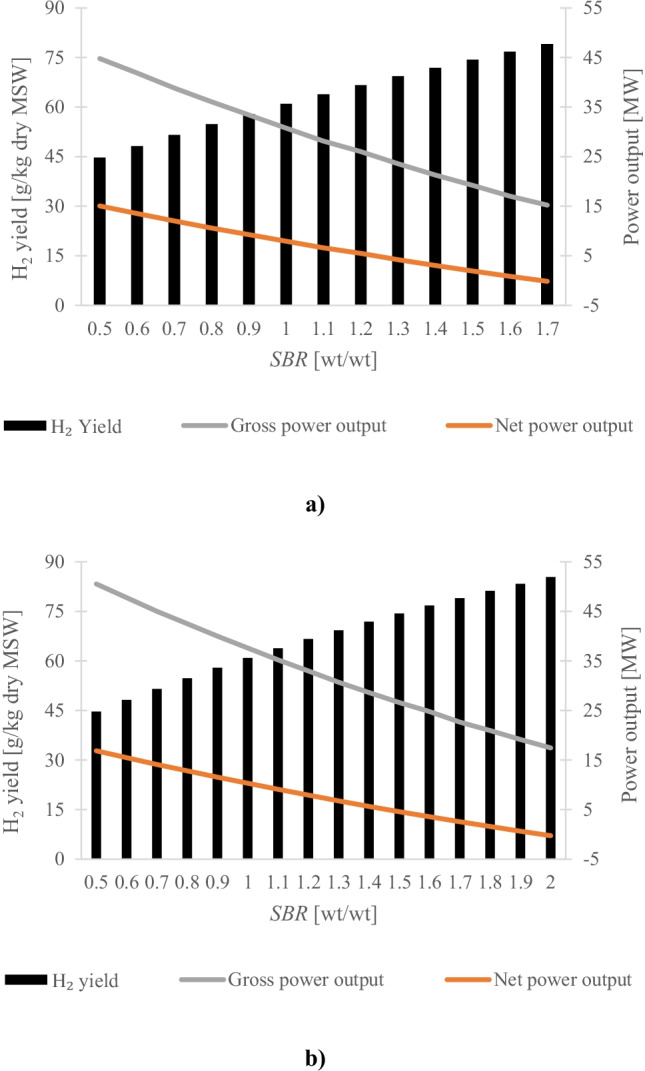
Effect of steam-to-biomass ratio, at gasification temperature of 650 °C, on hydrogen yield, gross and power outputs for sorption-enhanced gasification using (**a**) dolomite and (**b**) doped limestone with seawater as sorbent

A parametric study was carried out varying the steam-to-biomass ratio (*SBR*) between 0.5 and 1.7 and between 0.5 and 2.0 for SEG using dolomite and doped limestone, respectively. The *SBR* ranges are different because it was assumed that the plant is energetic self-sufficient, which after the last point of the interval (*SBR* = 1.7 and *SBR* = 2.0, for dolomite and doped limestone, respectively) is no more valid. The gasification temperature was kept constant at 650 °C because for temperatures behind 680 °C the CO_2_ capture is controlled by the equilibrium of carbonation reaction [26]. The effect of *SBR* on the H_2_ yield and gross and net power outputs is shown in Fig. 2. This analysis revealed a trade-off between H_2_ production and the net power output. It can be seen the profiles of the analysed variables are similar for both sorbents. The H_2_ yield increased gradually with the *SBR* increase, which is due to the equilibrium shift of steam-methane reforming and water gas shift reactions. As the H_2_O content increases, the equilibrium changes and the forward reaction is favoured, thus boosting the H_2_ production. On the other hand, the gross and net power outputs decreased which can be attributed to the higher power consumption for the H_2_ final product compression and CO_2_ compression, as well as higher energy consumption for steam production. Moreover, the power generation by the gas turbine and the steam cycle was penalised. This is due to the reduced availability of the tail gas, from the H_2_ upgrading unit, to be burnt in the gas turbine and the heat excess to be recovered. These trends were observed for both sorbents with some particularities. In the case of dolomite, the lower electric power generated by the gas turbine was compensated by the higher electric power generated by the steam cycle. It was because there was less tail gas available to be burnt in the gas turbine and there was more heat excess recovered in the steam cycle. The latter can be attributed to the fact that more solids were recirculated. Therefore, more heat was released at the carbonator, which is in agreement with the study carried out by Ortiz et al. [68] for CaL process. In the case of doped limestone, since less tail gas was needed to meet the energy requirement of the calciner, the electric power generated by the gas turbine compensated the lower one generated by the steam cycle.

**Fig. 3:**
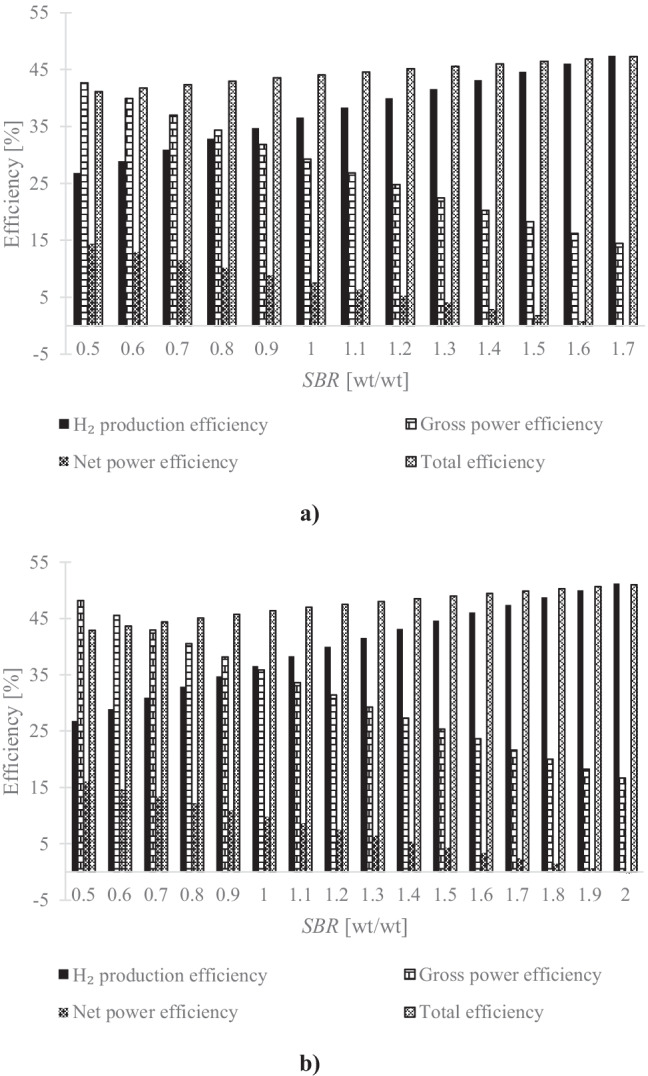
Effect of steam-to-biomass ratio, at gasification temperature of 650 °C, on hydrogen production, gross power, net power and total efficiencies for sorption-enhanced gasification using (**a)** dolomite and (**b)** doped limestone with seawater as sorbent

The effect of *SBR* on the thermodynamic performance indicators, described in Sect. 3, is presented in Fig. 3. As would be expected, the H_2_ production, gross power and net power efficiencies follow the trends seen for H_2_ yield, gross and net power outputs (Fig. 2). The total efficiency increased marginally in the *SBR* range investigated. Because this work intends to compare the H_2_ production from MSW SEG using different sorbents, to perform a fair comparison, the optimum *SBR* was determined as the value at which there was a change from positive to negative sign on the net power output. This means the plant is self-sufficient from an energy standpoint and the H_2_ production is maximised. Thus, the optimum *SBR* was 1.6 and 1.9 for dolomite and doped limestone, respectively. For higher SBR values, the electricity generated by the system was insufficient to meet the auxiliary power requirement of the SEG Process. Consequently, the SEG plant would need to draw electricity from the grid. At the optimum *SBR*, the SEG process was found to result in an H_2_ yield of 76.7 and 83.3 g/kg dry MSW (Fig. 2) for dolomite and doped limestone, respectively. This translated into an H_2_ production efficiency of 46.0% and 50.0% (Fig. 3). Similar to the H_2_ production efficiency, the use of dolomite resulted in a lower total efficiency by 4 percentage points, 46.8% (dolomite) compared to that of the SEG process using doped limestone (50.6%). Although the calciner temperature can be reduced from 900 to 850 °C when dolomite was used as sorbent, a higher sorbent make-up was fed to the calciner due to the presence of inert material. This result is in agreement with the reported for the CaL process [68]. Consequently, more tail gas from the H_2_ upgrading unit was consumed as fuel in the calciner and less was available for power generation in the gas turbine. Besides, more power was required by the ASU and the CO_2_ compression unit. The latter can be explained by the fact that more fresh material was calcined. At the carbonator operating conditions (650 °C), the MgO content in the dolomite is not carbonated and more fresh sorbent is needed. Therefore, the SEG process using dolomite has shown to have the highest heat requirement in the calciner (4.9 GJ/tCO_2_); on the other hand, the SEG process using doped limestone presents the lowest figure (4.6 GJ/tCO_2_).

### Economic performance

Because there is still some discrepancy between the economic data reported in the literature, the economic assessment was performed for different scenarios. In scenario 1, considered as the baseline scenario, there was no gate fee or fossil CO_2_ emissions tax considered; scenario 2 accounted for a gate fee but no tax on fossil CO_2_ emissions; in scenario 3, the levy of fossil CO_2_ emissions but without a gate fee was considered; and in scenario 4, the application of both, gate fee and fossil CO_2_ emissions tax, were considered.

In the techno-economic evaluation of scenario 2 and scenario 4, it was assumed that the SEG plant charges a fee of 40.0 €/t_MSW_, gate fee, to the waste disposers [67]. In scenario 3 and scenario 4, to estimate the fossil CO_2_ emissions, it was assumed there were no fluctuations in MSW composition over the year and 60% of the carbon present was of fossil origin [67]. It is worth noting that the CO_2_ emissions from fresh sorbent calcination were also accounted for in the calculation of fossil CO_2_ emissions [58]. These CO_2_ emissions are the only ones levied with the CO_2_ emission allowance price (EUA), which was estimated taking into account the average value for the first trimester of 2021, 39.6 €/tCO_2_ [66]. This is a conservative figure since the value in the current year of 2022 is close to 90.0 €/tCO_2_.

**Fig. 4:**
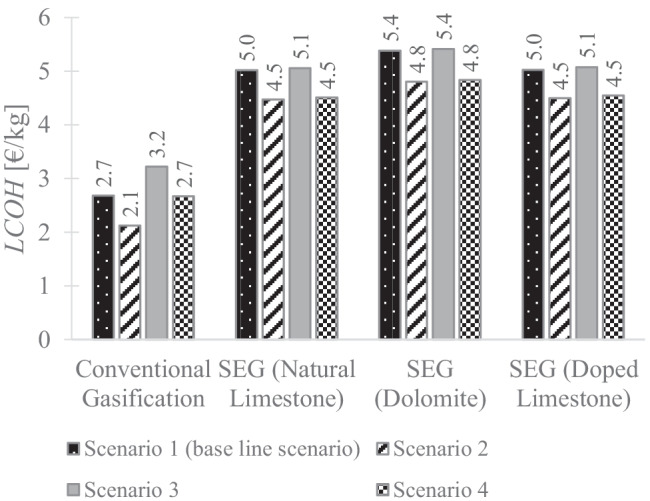
Comparison of levelised cost of hydrogen of conventional gasification and of sorption-enhanced gasification using natural limestone, dolomite and doped limestone with seawater as sorbent, for the different scenarios

**Fig. 5:**
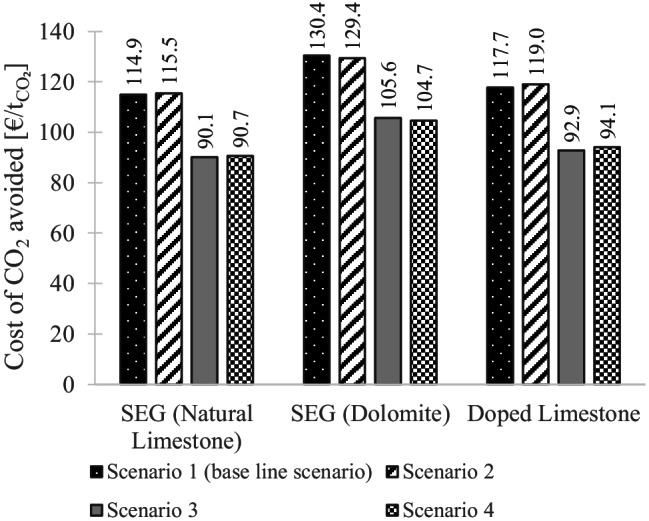
Comparison of cost of CO_2_ avoided for sorption-enhanced gasification using natural limestone, dolomite and doped limestone with seawater as sorbent, for the different scenarios

The estimated *LCOH* and CO_2_ avoided cost of SEG using dolomite and doped limestone for each scenario are depicted in Figs. 4 and 5, respectively. The *LCOH* of SEG is also compared with conventional gasification in Fig. 4. It can be seen from this figure that there is no difference in the trend observed for each sorbent over the scenarios. For both sorbents, the selection of SEG technology over gasification technology led to an increase in the *LCOH*. However, this rise ranged between 79.5 and 100.0% in the case of dolomite and for doped limestone felt between 67.9 and 89.2%. This can be attributed to the higher conversion in the carbonator obtained in the case of doped limestone and thus, a higher H_2_ production was achieved.

Regarding the baseline scenario (scenario 1) the *LCOH* increased from 2.7 (conventional gasification) to 5.4 or 5.0 €/kg of SEG using dolomite or SEG using doped limestone, respectively. These figures corresponded to a CO_2_ avoided cost of 130.4 and 117.7 €/tCO_2_ in the case of dolomite and doped limestone, respectively.

The application of a gate fee (scenario 2) decreased the *LCOH* of conventional gasification and SEG due to an additional revenue obtained by the waste management plant. While this reduction was more pronounced for conventional gasification, about 20.5%, in the case of SEG was about half of this figure for both sorbents, around 10.5%. Because both technologies benefited from this additional revenue, this difference was not reproduced on the cost of CO_2_ avoided (Fig. 5), which varied just about 1%.

On the other hand, in scenario 3, as more than 90.0% of the CO_2_ emissions were captured in the case of SEG, the levy of fossil CO_2_ emissions reduced the cost of CO_2_ avoided by around 20.0% (from 130.4 to 105.6 €/tCO_2_, in the case of dolomite and 177.7 to 92.9 €/tCO_2_, in the case of doped limestone) compared to baseline scenario 1. While the *LCOH* increased by 20.0% in the conventional gasification, the *LCOH* of SEG just increased by 0.6 and 0.8% in the case of dolomite and doped limestone, respectively (Fig. 4).

It can be observed in Fig. 4 that the application of both gate fee and fossil CO_2_ emissions tax (scenario 4) did not impact the *LCOH* of conventional gasification. This happened because the additional revenue obtained from the gate fee compensated the additional cost regarding the tax on fossil CO_2_ emissions. Nevertheless, the *LCOH* of SEG was reduced by 10.0% (from 5.4 and 5.0 €/kg to 4.8 and 4.5 €/kg, for dolomite and doped limestone, respectively). This corresponded to a decrease in the cost of CO_2_ avoided of around 20.0% when compared with the baseline Scenario 1 (130.4 against 104.7 and 117.7 against 94.1 €/tCO_2_, in the case of dolomite and doped limestone, respectively).

### Sensitivity analysis

**Fig. 6:**
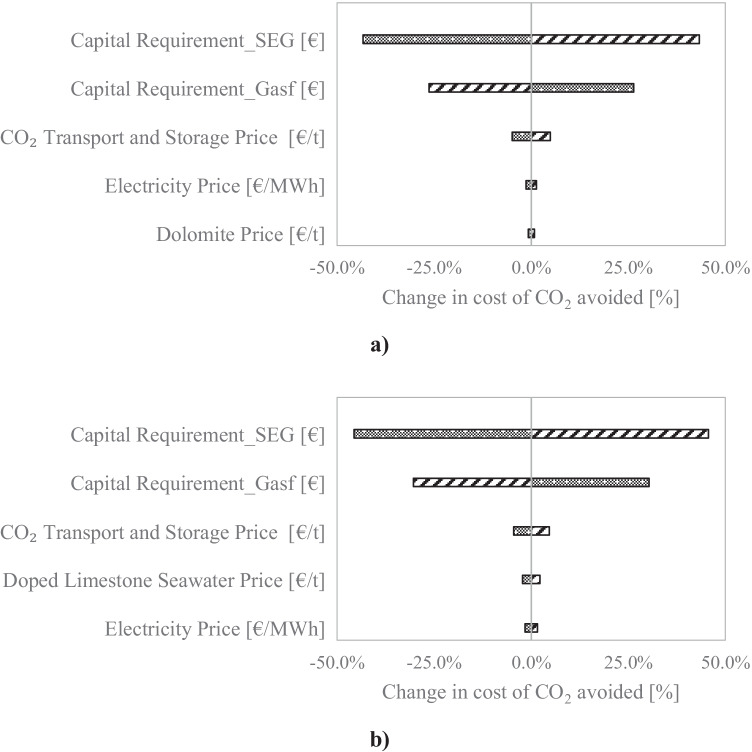
Effect of the main economic parameters on the cost of CO_2_ avoided: (**a)** using dolomite as sorbent and (**b)** using doped limestone with seawater as sorbent. Bubbles: − 25% of baseline parameter; stripes: + 25% of baseline parameter

Since there is still some uncertainty associated with the economic assessment and in particular to the capital cost of SEG, which is very scarce, a sensitivity analysis on the main economic parameters was performed. The effect of these parameters on the cost of CO_2_ avoided was investigated by varying their values by ± 25% for the baseline scenario 1 (Fig. 6). The conventional gasification and SEG capital requirements, in addition to the prices of CO_2_ transport and storage, electricity and sorbent were the parameters considered in the sensitivity analysis.

As can be seen in Fig. 6, the results are quite similar for both sorbents, with the capital requirement playing the main role in the cost of CO_2_ avoided. An increase of 25% in the capital cost of SEG led to a 43.3% and 45.6% increase in the cost of CO_2_ avoided for dolomite and doped limestone, respectively. On the other hand, the cost of CO_2_ avoided can be lowered by 26.3% and 30.3% for dolomite and doped limestone, respectively, if the capital required by conventional gasification rises by 25%. The only difference observed between the two sorbents stems from the difference in the sorbent cost. While the variation of ± 25% on dolomite price changed the cost of CO_2_ avoided by no more than ± 0.8%, the cost associated with the doped dolomite can influence the cost of CO_2_ avoided by ± 2.2%. This can be explained by the fact that it was assumed that the price of doped limestone (58.0 €/t) was 5 times the price of natural limestone (11.6 €/t), the latter was assumed to be the price of dolomite. For that reason, and since there is no precise cost associated with doping limestone, a further sensitivity for the baseline scenario (no gate fee or fossil CO_2_ emissions tax) was carried out.

**Fig. 7:**
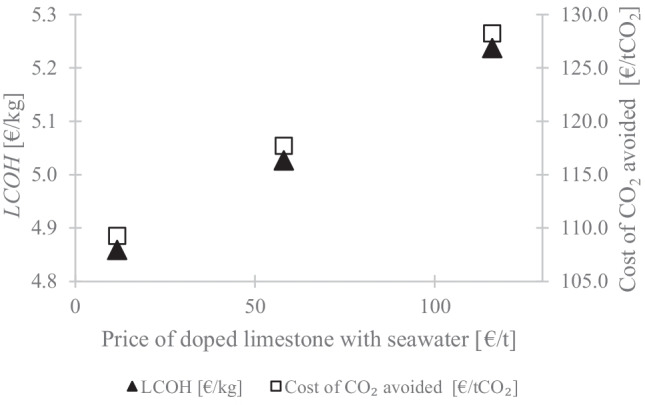
Effect of doped limestone price on the levelised cost of hydrogen and cost of CO_2_ avoided

To understand how the cost of doped limestone influences the SEG MSW viability, the effect of the doped limestone price on the *LCOH* and the cost of CO_2_ avoided was assessed. The price of the doped limestone was varied from 11.6 €/kg (limestone price) to 116 €/kg (10× limestone price). The results are shown in Fig. 7. It can be observed that the *LCOH* can vary from 4.9 to 5.2 €/kg, which corresponds to a cost of CO_2_ avoided between 109.3 and 128.4 €/tCO_2_. Thus, if the doping process did not impose any cost penalty on sorbent price, the use of doped limestone would slightly reduce the *LCOH* from 5.0 (natural limestone) to 4.9 €/kg, followed by a reduction of 5% on the cost of CO_2_ avoided.

The comparison of SEG performance using dolomite and doped limestone with that of SEG using natural limestone and conventional gasification is presented in Table 4. It should be mentioned that the optimum conditions for each case were a trade-off between H_2_ productivity and net power efficiency. Thus, in all cases, it was considered an energetic self-sufficient plant.

**Table 4:** Summary of techno-economic performance of conventional gasification and sorption-enhanced gasification. The latter was carried out for three sorbents: limestone, dolomite and doped limestone with seawater

Parameter	Conventional gasification	Sorption-enhanced gasification		
		Natural limestone	Dolomite	Doped limestone
Thermodynamic assessment				
H_2_ production efficiency (%)	47.7	48.7	46.0	50.0
Gross power efficiency (%)	18.9	17.0	16.2	18.3
Net power efficiency (%)	6.0	0.6	0.8	0.6
Total efficiency (%)	53.3	49.3	46.8	50.6
Environmental assessment				
Equivalent CO_2_ emissions ($${\mathbf{kg}}_{\mathbf{C}{\mathbf{O}}_{\mathbf{2}}}$$$${\mathbf{kg}}_{{\mathbf{H}}_{\mathbf{2}}}$$)	21.7	1.4	1.0	1.8
Economic assessment				
Levelised cost of H_2_ (€/kg)	2.7	5.0	5.4	5.0
Cost of CO_2_ avoided (€/tCO_2_)		114.9	130.4	117.7

Regarding the thermodynamic performance, the technology SEG using doped limestone presented a higher H_2_ production efficiency, 50.0%. This can be attributed to the enhanced CO_2_ sorption capacity of doped limestone. When compared with conventional gasification, this higher H_2_ productivity was obtained at the expense of electrical power production. The net power efficiency of conventional gasification (6.0%) was more from 5 percentage points than that of SEG using doped limestone (0.6%).

As can be seen from Table 4, the integration of CO_2_ capture, SEG, led to a reduction of more than 90% of equivalent CO_2_ emissions. These decreased from 21.7 ${\mathsf{kg}}_{\mathsf{C}{\mathsf{O}}_{\mathsf{2}}}$/${\mathsf{kg}}_{{\mathsf{H}}_{\mathsf{2}}}$. (conventional gasification) to the range between 1.0 and 1.8 ${\mathsf{kg}}_{\mathsf{C}{\mathsf{O}}_{\mathsf{2}}}$/${\mathsf{kg}}_{{\mathsf{H}}_{\mathsf{2}}}$.

The economic assessment has shown that the introduction of CO_2_ capture doubles the *LCOH* when dolomite was used as a sorbent. Between the natural and doped limestone, the former presented a lower cost of CO_2_ avoided (114.9 €/tCO_2_) than the latter (117.7 €/tCO_2_). It is noteworthy that in the analysis of doped limestone, the energy penalty associated with sorbent drying was associated with the higher sorbent price. However, it is clear from Fig. 7 that if the cost of doped limestone is reduced to below 42.6 €/t, this sorbent would present the lowest cost of CO_2_ avoided. Thus, the doped limestone seems to be a route that should be explored to replace natural limestone, whose one of the main drawbacks is the deactivation over the cycles.

## Conclusions

In this work, the techno-economic performance of MSW SEG using three CaO-based sorbents, natural limestone, dolomite and doped limestone, was compared. While the H_2_ production was one of the key thermodynamic performance indicators selected, the *LCOH* and cost of CO_2_ avoided were the key economic performance indicators. The use of limestone as sorbent has shown to have the best techno-economic performance. The *LCOH* of SEG using dolomite is 5.4 €/kg against 5.0 €/kg when the limestone is used as sorbent. The natural limestone has shown to have the lowest cost of CO_2_ avoided (114.9 €/tCO_2_), whereas the doped limestone had the highest H_2_ production efficiency (50.0%). In this work, the heat requirement in the calciner falls between 4.6 GJ/tCO_2_ (doped limestone) and 4.9 GJ/tCO_2_ (dolomite). These results revealed a link between the heat requirement in the calciner and the H_2_ production costs; however, detailed work on the effect of calcination temperature on energy requirement and overall costs should be carried out. A sensitivity analysis on the cost of CO_2_ avoided was also performed by varying the main economic parameters by ± 25%. The capital requirement of conventional gasification and SEG, the CO_2_ transport and storage price, the electricity and sorbent prices were the selected parameters in the sensitivity analysis. From these parameters, the capital requirement has the greatest influence on the CO_2_ avoided cost, a reduction of 25% of SEG capital cost reduced the cost of CO_2_ avoided by more than 40%. Although there is no data available for doped limestone price, it was found that a reduction on its price to below 42.6 €/t would reduce the cost of CO_2_ avoided to a value lower than that for natural limestone. Furthermore, new sorbents for CO_2_ capture and H_2_ production would be an attractive alternative to natural limestone if produced with similar costs to that of natural limestone.

## Supplementary Information

ESM 1 (DOCX 32.4 kb)

## Nomenclature

*a*_1_, *a*_2_: fitting constant of sorbent maximum average conversion model (-)
*A*_*j*_: heat exchanger area of equipment j (m^2^)
*AC*: cost of CO_2_ avoided (€/tCO_2_)
ASU: air separation unit
*b*: fitting constant of sorbent maximum average conversion model (-)
BFB: bubbling fluidised bed
CCS: carbon capture and storage
CEPCI: Chemical Engineering Plant Cost Index
*e*_CO2,eq_: equivalent CO_2_ emissions (kgCO_2_/kgH_2_)
EUA: CO_2_ emission allowance price (€/tCO_2_)
*f*_1,_*f*_2_: fitting constant of sorbent maximum average conversion model (-)
*f*_*i*_: reaction extent (-)
*F*_0_: fresh make-up sorbent rate (kmol/s)
*F*_R_: sorbent circulation rate (kmol/s)
*LCOH*: levelised cost of hydrogen (€/kg)
*LHV*: lower heating value (MJ/kg)
${\dot{m}}_i$: flowrate of component *i* (kg/s) or (t/h) or (kg/h)
MSW: municipal solid waste
*NPV*: net present value (€)
PSA: pressure swing adsorption
*SBR*: steam-to-biomass ratio (kg/kg)
SEG: sorption-enhanced gasification
TGA: thermogravimetric analyser
*W*_el, gross_: gross electric power output (MW_el_)
*W*_el, net_: net electric power output (MW_el_)
$\dot{W_{\boldsymbol{j}}}$: brake power requirement/output of equipment *j* (kW_el_)

## Greek letters

$${\eta}_{{\mathrm{H}}_2}$$: H_2_ production efficiency
*η*_el, gross_: gross power efficiency
*η*_el, net_: net power efficiency
*η*_el, net_: total efficiency

## Subscripts

ASU: air separation unit
BRKP: brake power
calc: calcination
carb: carbonation
CCU: CO_2_ compression unit
COND: condensate
CW: freshwater
DEA: deaerator
eq: equivalent
ECON: economiser
FC: fuel compressor
Gasf: conventional gasification
GT: gas turbine
HPST: high-pressure steam turbine
HPW: high-pressure water
HRSG: heat recovery steam generator
IPST: intermediate-pressure steam turbine
LPST: low-pressure steam turbine
LPW: low-pressure water
LS: live steam
