# Supplementary material for: Sorption-enhanced gasification of municipal solid waste for hydrogen production: a comparative techno-economic analysis using limestone, dolomite and doped limestone
Source: Biomass Convers Biorefin. 2022 Jun 23:1–16. Online ahead of print. doi: 10.1007/s13399-022-02926-y (PMC9219401; doi:10.1007/s13399-022-02926-y)
Supplement: Supplementary file 1 — (DOCX 32.4 kb) [file 13399_2022_2926_MOESM1_ESM.docx]

Supplementary information

Table A1. Cost correlations used on the estimation of the capital cost of each unit

| Unit operation | Cost Correlation |
| --- | --- |
| *Sorption enhanced gasification* |  |
| Sorption enhanced gasifier [Installed capacity LHV, *P_inst_* (MW)] [8] | $\text{C}_{\text{SEG}}\text{ =22.1e6 }\left( \frac{\text{P}_{\text{inst}}}{\text{10}} \right)^{\text{0.80}}$ |
| Calciner [Calciner heat flux, ${\dot{\text{Q}}}_{\boldsymbol{calc}}$ (kW_th_)] [69] | $\text{C}_{\text{calc}}\text{ = 13140} {\text{(}{\dot{\text{Q}}}_{\text{cal}}\text{)}}^{\text{0.67}}$ |
| Air separation unit [O_2_ production rate, ${\dot{\text{m}}}_{\text{O}_{\text{2}}}$(kg/s)] [70] | $\text{C}_{\text{ASU}}\text{ = 2.926e7}\left( \frac{{\dot{\text{m}}}_{\text{O}_{\text{2}}}}{\text{28.9}} \right)^{\text{0.70}}$ |
| *H_2_-rich syngas upgrading* |  |
| Pressure swing adsorption unit [Inlet gas molar flowrate,${\dot{\text{n}}}_{\text{PSA}}$ *(*kmol/h)] [71] | $\text{C}_{\text{PSA}}\text{ = 27.96e6}\left( \frac{{\dot{\text{n}}}_{\text{PSA}}}{\text{17069}} \right)^{\text{0.60}}$ |
| H_2_ compressor [Brake power requirement, ${\dot{\text{W}}}_{\text{H}_{\text{2}}\text{,BRK}}$ *(*kW_el_)] [71] | $\text{C}_{\text{H}_{\text{2}}\text{Comp}}\text{ =1200}\left( \frac{{\dot{\text{W}}}_{\text{H}_{\text{2}}\text{,BRK}}}{\text{0.746}} \right)^{\text{0.82}}$ |
| *CO_2_ compression* |  |
| CO_2_ compression unit [Brake power requirement, ${\dot{\text{W}}}_{\text{CCU,}\text{BRK}}$(kW_el_)] [72] | $\text{C}_{\text{CCU}}\text{ = 1.22914e7}\left( \frac{{\dot{\text{W}}}_{\text{CCU,BRK}}}{\text{13000}} \right)^{\text{0.67}}$ |
| Fuel compressor [Brake power requirement, ${\dot{\text{W}}}_{\text{FC,}\text{BRK}}$(kW_el_)] [73, 74] | $\text{C}_{\text{FC}}\text{ = 91562}\left( \frac{{\dot{\text{W}}}_{\text{FC,BRK}}}{\text{445}} \right)^{\text{0.67}}$ |
| *Steam Cycle* |  |
| High-pressure steam turbine [Brake power output, *Ẇ_HPST_,_BRK_* (kW_el_)] [75] | $\text{C}_{\text{HPST}}\text{ = 33.7e6}\left( \frac{{\dot{\text{W}}}_{\text{HPST,BRK}}}{\text{200000}} \right)^{\text{0.67}}$ |
| Intermediate-pressure steam turbine [Brake power output, *Ẇ_IPST_,_BRK_* (kW_el_)] [75] | $\text{C}_{\text{IPST}}\text{ = 33.7e6}\left( \frac{{\dot{\text{W}}}_{\text{IPST,BRK}}}{\text{200000}} \right)^{\text{0.67}}$ |
| Low-pressure steam turbine [Brake power output, *Ẇ_LPST_,_BRK_* (kW_el_)] [75] | $\text{C}_{\text{LPST}}\text{ = 33.7e6}\left( \frac{{\dot{\text{W}}}_{\text{LPST,BRK}}}{\text{200000}} \right)^{\text{0.67}}$ |
| Deaerator [Inlet flowrate, ${\dot{\text{m}}}_{\text{DEA }}$(kg/h)] [76] | $\text{C}_{\text{DEA}}\text{ = 1.30721e5}\left( \frac{{\dot{\text{m}}}_{\text{DEA }}}{\text{157970.7}} \right)^{\text{0.72}}$ |
| Deaerator feed pump [Condensate flowrate, ${\dot{\text{m}}}_{\text{COND }}$(kg/h)] [76] | $\text{C}_{\text{P\_DEA}}\text{ = 8679}\left( \frac{{\dot{\text{m}}}_{\text{COND}}}{\text{158425.2}} \right)^{\text{0.33}}$ |
| Low-pressure water pump [Feed water flowrate, ${\dot{\text{m}}}_{\text{LPW }}$(kg/h)] [76] | $\text{C}_{\text{P\_LPW}}\text{ = 95660}\left( \frac{{\dot{\text{m}}}_{\text{LPW}}}{\text{158425.2}} \right)^{\text{0.33}}$ |
| Fresh water pump [Fresh water flowrate, ${\dot{\text{m}}}_{\text{CW }}$(kg/h)] [76] | $\text{C}_{\text{P\_CW}}\text{ = 5437}\left( \frac{{\dot{\text{m}}}_{\text{CW }}}{\text{42625.9}} \right)^{\text{0.33}}$ |
| Heat exchanger H_2_-rich syngas cooler/Steam generator [PINCH, ${{\dot{\text{Q}}}_{\text{Syngas-SG}}}_{\text{ }}$(kW_th_)] [76] | $\text{C}_{\text{Syngas-SG}}\text{ = 26143}\left( \frac{{\dot{\text{Q}}}_{\text{Syngas-SG}}}{\text{401.5}} \right)^{\text{0.60}}$ |
| Condenser [Heat exchange area, *A_COND_* (m^2^)] [77] | $\text{C}_{\text{COND}}\text{ = 8500+490}{\text{(}\text{A}_{\text{COND}}\text{)}}^{\text{0.85}}$ |
| Heat exchanger live steam [Heat exchange area, *A_LS_* (m^2^)] [74] | $\text{C}_{\text{LS}}\text{ = 2290}\left( \text{A}_{\text{LS}} \right)^{\text{0.60}}$ |
| Heat exchanger condensate [Heat exchange area, *A_COND_* (m^2^)] [73] | $\text{C}_{\text{COND}}\text{ = 130}\left( \frac{\text{A}_{\text{COND}}}{\text{0.093}} \right)$ |
| Heat recovery steam generator [Steam flowrate, *Q_HRSG_* (kg/h)] [78] | $\text{C}_{\text{HRSG}}\text{ =42427}\left( \frac{\text{Q}_{\text{HRSG}}}{\text{277458}} \right)^{\text{0.7}}$ |
| Heat exchanger high-pressure water [Heat exchange area, *A_HPW_* (m^2^)] [73] | $\text{C}_{\text{HPW}}\text{ = 130}\left( \frac{\text{A}_{\text{HPW}}}{\text{0.093}} \right)$ |
| Economiser [Heat exchange area, *A_ECON_* (m^2^)] [73] | $\text{C}_{\text{ECON}}\text{ = 130}\left( \frac{\text{A}_{\text{ECON}}}{\text{0.093}} \right)$ |
| *Gas turbine* |  |
| Gas turbine [Inlet air flowrate, ${\dot{\text{m}}}_{\text{Air}}$ (kg/s)] [71] | $\text{C}_{\text{GT}}\text{ = 31.5e6}\left( \frac{{\dot{\text{m}}}_{\text{Air}}}{\text{209}} \right)^{\text{0.85}}$ |
| *Pre-treatment* |  |
| Pre-treatment [Processing capacity, ${\dot{\text{m}}}_{\text{MSW }}$(t/h)] [51] | $\text{C}_{\text{Pre-t}}\text{ =}\left( \text{9.0e4}{\dot{\text{m}}}_{\text{MSW}}\text{+6.6e4} \right)\text{+}\left( \text{7.1e4}{\dot{\text{m}}}_{\text{MSW}}\text{+8.0e4} \right)$ |

References

69. Michalski S, Hanak DP, Manovic V (2019) Techno-economic feasibility assessment of calcium looping combustion using commercial technology appraisal tools. Journal of Cleaner Production 219:540–551. <https://doi.org/10.1016/j.jclepro.2019.02.049>

70. Atsonios K, Koumanakos A, Panopoulos KD, et al (2013) Techno-economic comparison of CO_2_ capture technologies employed with natural gas derived GTCC. In: Proceedings of the ASME Turbo Expo. p V002T07A018. <https://doi.org/10.1115/GT2013-95117>

71. Spallina V, Pandolfo D, Battistella A, et al (2016) Techno-economic assessment of membrane assisted fluidized bed reactors for pure H_2_ production with CO_2_ capture. Energy Conversion and Management 120:257–273. <https://doi.org/10.1016/j.enconman.2016.04.073>

72. Kreutz T, Williams R, Consonni S, Chiesa P (2005) Co-production of hydrogen, electricity and CO from coal with commercially ready technology. Part B: Economic analysis. International Journal of Hydrogen Energy 30:769–784. <https://doi.org/10.1016/j.ijhydene.2004.08.001>

73. Lee YD, Ahn KY, Morosuk T, Tsatsaronis G (2014) Exergetic and exergoeconomic evaluation of a solid-oxide fuel-cell-based combined heat and power generation system. Energy Conversion and Management 85:154–164. <https://doi.org/10.1016/j.enconman.2014.05.066>

74. Shirazi A, Aminyavari M, Najafi B, et al (2012) Thermal–economic–environmental analysis and multi-objective optimization of an internal-reforming solid oxide fuel cell–gas turbine hybrid system. International Journal of Hydrogen Energy 37:19111–19124. <https://doi.org/10.1016/j.ijhydene.2012.09.143>

75. Manzolini G, Macchi E, Gazzani M (2013) CO_2_ capture in natural gas combined cycle with SEWGS. Part B: Economic assessment. International Journal of Greenhouse Gas Control 12:502–509. <https://doi.org/10.1016/j.ijggc.2012.06.021>

76. NREL (2005) Biomass to Hydrogen Production Detailed Design and Economics Utilizing the Battelle Columbus Laboratory Indirectly-Heated Gasifier. Technical Report NREL/TP-510-37408

77. Sayyaadi H, Mehrabipour R (2012) Efficiency enhancement of a gas turbine cycle using an optimized tubular recuperative heat exchanger. Energy 38:362–375. <https://doi.org/10.1016/j.energy.2011.11.048>

78. NETL (2019) Cost and performance baseline for fossil energy plants. Volume 1: Bituminous coal and natural gas to electricity. Technical Report NETL-PUB-22638
